# Bis{2-[(*E*)-benzyl­imino­meth­yl]-4-methyl­phen­olato-κ^2^ *N*,*O*}cobalt(II)

**DOI:** 10.1107/S1600536808034144

**Published:** 2008-11-08

**Authors:** Fang-Fang Dang, Xin-Wei Wang, Yuan-Zhen Zhou, Guo-Ping Han, Qing-Cui Yang

**Affiliations:** a School of Science, Xi’an University of Architecture and Technology, Xi’an 710055, People’s Republic of China; b Xi’an LiBang Pharmaceutical Co. Ltd, Xi’an 710086, People’s Republic of China

## Abstract

In the title complex, [Co(C_15_H_14_NO)_2_], the Co^II^ atom, situated on an inversion centre, is coordinated by two O and two N atoms from two symmetry-related bidentate Schiff base ligands in a slightly distorted square-planar geometry. The two phenolate rings form a dihedral angle of 10.53 (2)°.

## Related literature

For background on complexes of Schiff bases with transition metals, see: Rodriguez Barbarin *et al.* (1994 ▶).
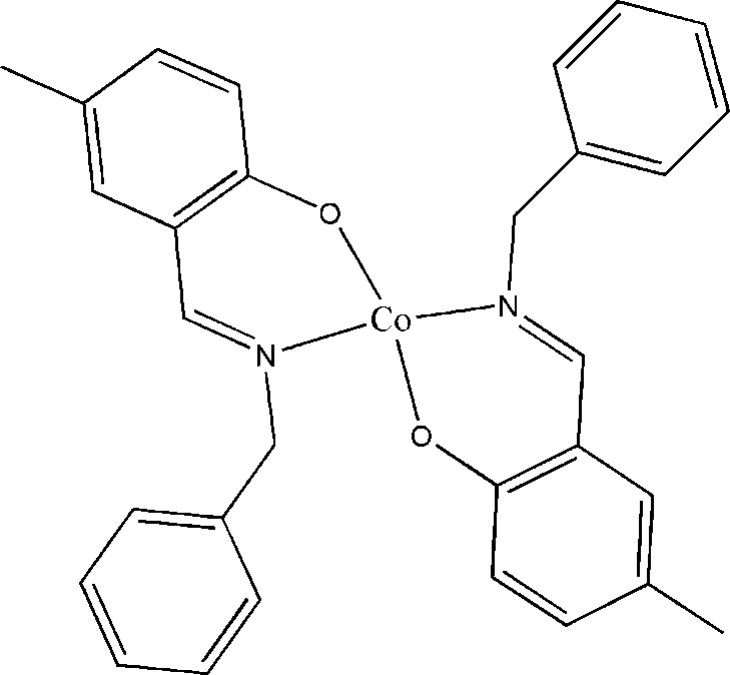


## Experimental

### 

#### Crystal data

[Co(C_15_H_14_NO)_2_]
*M* *_r_* = 507.47
Monoclinic, 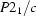

*a* = 13.735 (3) Å
*b* = 10.625 (2) Å
*c* = 8.7926 (17) Å
β = 107.394 (2)°
*V* = 1224.5 (4) Å^3^
*Z* = 2
Mo *K*α radiation
μ = 0.73 mm^−1^
*T* = 296 (2) K
0.37 × 0.30 × 0.25 mm

#### Data collection

Bruker SMART APEXII diffractometer
Absorption correction: multi-scan (*SADABS*; Sheldrick, 1997 ▶) *T*_min_ = 0.765, *T*_max_ = 0.825
10315 measured reflections
2807 independent reflections
2404 reflections with *I* > 2σ(*I*)
*R*_int_ = 0.022

#### Refinement

*R*[*F* ^2^ > 2σ(*F* ^2^)] = 0.027
*wR*(*F* ^2^) = 0.080
*S* = 1.08
2807 reflections
161 parameters
H-atom parameters constrained
Δρ_max_ = 0.22 e Å^−3^
Δρ_min_ = −0.24 e Å^−3^

### 

Data collection: *APEX2* (Bruker, 2004 ▶); cell refinement: *SAINT* (Bruker, 2004 ▶); data reduction: *SAINT*; program(s) used to solve structure: *SHELXS97* (Sheldrick, 2008 ▶); program(s) used to refine structure: *SHELXL97* (Sheldrick, 2008 ▶); molecular graphics: *XP* in *SHELXTL* (Sheldrick, 2008 ▶); software used to prepare material for publication: *XP* in *SHELXTL*.

## Supplementary Material

Crystal structure: contains datablocks global, I. DOI: 10.1107/S1600536808034144/gw2053sup1.cif

Structure factors: contains datablocks I. DOI: 10.1107/S1600536808034144/gw2053Isup2.hkl

Additional supplementary materials:  crystallographic information; 3D view; checkCIF report

## Figures and Tables

**Table:** 

Co1—O1	1.8259 (11)
Co1—N1	1.9258 (11)

**Table:** 

O1^i^—Co1—O1	180.0
O1^i^—Co1—N1	86.99 (5)
O1—Co1—N1	93.01 (5)

Symmetry code: (i) 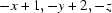.

**Fig. 1.**
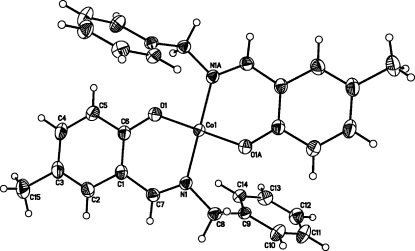
The structure of (I), showing displacement ellipsoids drawn at the 30% probability level. [Symmetry code: (A) -x + 1, -y + 2, -z]

## supplementary crystallographic information

### Comment

Schiff bases have played an important role in the development of coordination chemistry as they readily form stable complexes with most of the transition metals (Rodriguez Barbarin *et al.*, 1994). Salicylaldehyde and its derivatives are useful carbonyl precursors for the synthesis of a large variety of Schiff bases. Here we report on a new cobalt(II) complex (I).

The molecular structure of (I) as illustrated in Fig. 1 has the Co^2+^ center in a square geometry as it its coordinated by two O atoms and two N atoms from two 2-((*E*)-(benzylimino)methyl)-4-methylphenol bidentate chelating ligand. The Co1—O1 distance of 1.8259 (11) Å is shorter than the distance of Co1—N1 (1.9258 (11)) (Table 1). The dihedral angle between the plane of O1, N1, Co^2+^ and two parallel phenol rings with the distance of 0.484 Å is 10.53 °.

### Experimental

1 mmol of CoCl_2_.6H_2_O (0.238 g) were added to a 15 ml ethanol solution containing 2 mmol (0.450 g) 2-((*E*)-(benzylimino)methyl)-4-methylphenol. The resulting mixture was stirred for about 0.5 h. The slow vaporization of the solvent yielded after about 5 d dark brown single crystals. Yield: 68.8%. Calcd. for C_30_H_28_CoN_2_O_2_: C,71.00; H, 5.56; N, 5.52; Found: C, 71.31; H, 5.60; N,5.47%.

### Refinement

All H atoms were located from difference Fourier syntheses, H atoms from the C—H groups were placed in geometrically idealized positions and constrained to ride on their parent atoms (C—H = 0.93%A, 0.96%A, 0.97%A;) and *U*_iso_(H) values equal to 1.2 *U*_eq_(C).

### Crystal data

**Table:**

[Co(C_15_H_14_NO)_2_]	*F*_000_ = 530.0
*M**_r_* = 507.47	*D*_x_ = 1.376 Mg m^−^^3^
Monoclinic, *P*2_1_/*c*	Mo *K*α radiation λ = 0.71073 Å
Hall symbol: -P 2ybc	Cell parameters from 2457 reflections
*a* = 13.735 (3) Å	θ = 1.0–27.6º
*b* = 10.625 (2) Å	µ = 0.73 mm^−^^1^
*c* = 8.7926 (17) Å	*T* = 296 (2) K
β = 107.394 (2)º	Block, dark brown
*V* = 1224.5 (4) Å^3^	0.37 × 0.30 × 0.25 mm
*Z* = 2	

### Data collection

**Table:**

Bruker SMART APEXII diffractometer	2807 independent reflections
Radiation source: fine-focus sealed tube	2404 reflections with *I* > 2σ(*I*)
Monochromator: graphite	*R*_int_ = 0.022
*T* = 296(2) K	θ_max_ = 27.6º
φ and ω scans	θ_min_ = 1.0º
Absorption correction: multi-scan(SADABS; Sheldrick, 1997)	*h* = −17→17
*T*_min_ = 0.765, *T*_max_ = 0.825	*k* = −11→13
10315 measured reflections	*l* = −11→11

### Refinement

**Table:**

Refinement on *F*^2^	Secondary atom site location: difference Fourier map
Least-squares matrix: full	Hydrogen site location: inferred from neighbouring sites
*R*[*F*^2^ > 2σ(*F*^2^)] = 0.027	H-atom parameters constrained
*wR*(*F*^2^) = 0.080	*w* = 1/[σ^2^(*F*_o_^2^) + (0.0449*P*)^2^ + 0.166*P*] where *P* = (*F*_o_^2^ + 2*F*_c_^2^)/3
*S* = 1.08	(Δ/σ)_max_ < 0.001
2807 reflections	Δρ_max_ = 0.22 e Å^−^^3^
161 parameters	Δρ_min_ = −0.24 e Å^−^^3^
Primary atom site location: structure-invariant direct methods	Extinction correction: none

### Special details

**Table:**

Geometry. All e.s.d.'s (except the e.s.d. in the dihedral angle between two l.s. planes) are estimated using the full covariance matrix. The cell e.s.d.'s are taken into account individually in the estimation of e.s.d.'s in distances, angles and torsion angles; correlations between e.s.d.'s in cell parameters are only used when they are defined by crystal symmetry. An approximate (isotropic) treatment of cell e.s.d.'s is used for estimating e.s.d.'s involving l.s. planes.
Refinement. Refinement of *F*^2^ against ALL reflections. The weighted *R*-factor *wR* and goodness of fit *S* are based on *F*^2^, conventional *R*-factors *R* are based on *F*, with *F* set to zero for negative *F*^2^. The threshold expression of *F*^2^ > σ(*F*^2^) is used only for calculating *R*-factors(gt) *etc*. and is not relevant to the choice of reflections for refinement. *R*-factors based on *F*^2^ are statistically about twice as large as those based on *F*, and *R*- factors based on ALL data will be even larger.

### Fractional atomic coordinates and isotropic or equivalent isotropic displacement parameters (Å^2^)

**Table:**

	*x*	*y*	*z*	*U*_iso_*/*U*_eq_	
Co1	0.5000	1.0000	0.0000	0.03135 (9)	
N1	0.50882 (8)	0.85049 (10)	0.12722 (13)	0.0361 (2)	
O1	0.63563 (8)	1.03507 (11)	0.08454 (14)	0.0505 (3)	
C9	0.35213 (9)	0.85593 (12)	0.21649 (15)	0.0350 (3)	
C1	0.69440 (10)	0.84697 (13)	0.23467 (16)	0.0398 (3)	
C8	0.41511 (10)	0.78371 (12)	0.13166 (16)	0.0384 (3)	
H8A	0.4341	0.7033	0.1845	0.046*	
H8B	0.3735	0.7670	0.0233	0.046*	
C7	0.59332 (10)	0.80109 (13)	0.21412 (16)	0.0398 (3)	
H7A	0.5877	0.7280	0.2691	0.048*	
C14	0.39142 (11)	0.95166 (14)	0.32390 (16)	0.0402 (3)	
H14A	0.4589	0.9767	0.3425	0.048*	
C2	0.77878 (11)	0.77682 (15)	0.32681 (18)	0.0486 (3)	
H2A	0.7671	0.7011	0.3715	0.058*	
C6	0.71010 (11)	0.96291 (15)	0.16800 (17)	0.0408 (3)	
C3	0.87726 (11)	0.81665 (17)	0.35251 (19)	0.0534 (4)	
C10	0.25147 (11)	0.82053 (16)	0.19115 (18)	0.0501 (4)	
H10A	0.2237	0.7565	0.1192	0.060*	
C4	0.89188 (11)	0.93208 (18)	0.2855 (2)	0.0546 (4)	
H4A	0.9581	0.9612	0.3018	0.065*	
C11	0.19180 (12)	0.87956 (19)	0.2719 (2)	0.0608 (4)	
H11A	0.1244	0.8545	0.2541	0.073*	
C13	0.33122 (14)	1.01077 (14)	0.4041 (2)	0.0495 (4)	
H13A	0.3586	1.0751	0.4759	0.059*	
C12	0.23131 (14)	0.97510 (17)	0.3785 (2)	0.0554 (4)	
H12A	0.1910	1.0149	0.4322	0.066*	
C5	0.81198 (13)	1.00369 (15)	0.1967 (2)	0.0511 (4)	
H5A	0.8250	1.0800	0.1548	0.061*	
C15	0.96691 (15)	0.7397 (2)	0.4506 (3)	0.0824 (6)	
H15A	0.9442	0.6822	0.5171	0.124*	
H15B	1.0177	0.7949	0.5161	0.124*	
H15C	0.9957	0.6933	0.3808	0.124*	

### Atomic displacement parameters (Å^2^)

**Table:**

	*U*^11^	*U*^22^	*U*^33^	*U*^12^	*U*^13^	*U*^23^
Co1	0.02864 (14)	0.03280 (15)	0.03417 (14)	0.00058 (9)	0.01176 (10)	0.00084 (9)
N1	0.0347 (5)	0.0355 (6)	0.0411 (6)	−0.0002 (4)	0.0158 (5)	−0.0030 (5)
O1	0.0353 (5)	0.0509 (6)	0.0613 (7)	−0.0012 (4)	0.0085 (5)	0.0136 (5)
C9	0.0351 (6)	0.0364 (7)	0.0347 (6)	−0.0026 (5)	0.0120 (5)	0.0031 (5)
C1	0.0369 (7)	0.0438 (7)	0.0403 (7)	0.0051 (5)	0.0143 (5)	−0.0024 (6)
C8	0.0393 (7)	0.0329 (6)	0.0447 (7)	−0.0037 (5)	0.0150 (6)	−0.0022 (5)
C7	0.0430 (7)	0.0356 (7)	0.0437 (7)	0.0034 (5)	0.0173 (6)	0.0003 (5)
C14	0.0383 (7)	0.0410 (7)	0.0426 (7)	−0.0049 (6)	0.0144 (6)	−0.0023 (6)
C2	0.0454 (8)	0.0500 (8)	0.0520 (8)	0.0105 (6)	0.0169 (7)	0.0052 (7)
C6	0.0361 (7)	0.0466 (7)	0.0403 (7)	0.0034 (6)	0.0126 (6)	0.0003 (6)
C3	0.0395 (8)	0.0686 (10)	0.0526 (9)	0.0140 (7)	0.0146 (7)	0.0047 (8)
C10	0.0412 (8)	0.0631 (10)	0.0476 (8)	−0.0144 (7)	0.0158 (6)	−0.0118 (7)
C4	0.0332 (7)	0.0732 (12)	0.0589 (9)	0.0031 (7)	0.0162 (7)	0.0012 (8)
C11	0.0390 (8)	0.0865 (13)	0.0625 (10)	−0.0105 (8)	0.0237 (7)	−0.0095 (9)
C13	0.0578 (10)	0.0470 (9)	0.0475 (8)	−0.0029 (7)	0.0215 (8)	−0.0085 (6)
C12	0.0531 (10)	0.0660 (10)	0.0556 (9)	0.0064 (8)	0.0292 (8)	−0.0027 (8)
C5	0.0378 (8)	0.0589 (10)	0.0574 (10)	−0.0016 (6)	0.0157 (7)	0.0068 (7)
C15	0.0461 (9)	0.1026 (17)	0.0950 (14)	0.0244 (11)	0.0154 (10)	0.0311 (14)

### Geometric parameters (Å, °)

**Table:**

Co1—O1^i^	1.8259 (11)	C2—C3	1.370 (2)
Co1—O1	1.8259 (11)	C2—H2A	0.9300
Co1—N1	1.9258 (11)	C6—C5	1.414 (2)
Co1—N1^i^	1.9258 (11)	C3—C4	1.401 (2)
N1—C7	1.2946 (17)	C3—C15	1.514 (2)
N1—C8	1.4804 (16)	C10—C11	1.385 (2)
O1—C6	1.3129 (19)	C10—H10A	0.9300
C9—C14	1.3831 (19)	C4—C5	1.371 (2)
C9—C10	1.3851 (18)	C4—H4A	0.9300
C9—C8	1.5096 (17)	C11—C12	1.378 (3)
C1—C6	1.408 (2)	C11—H11A	0.9300
C1—C2	1.412 (2)	C13—C12	1.376 (2)
C1—C7	1.4311 (19)	C13—H13A	0.9300
C8—H8A	0.9700	C12—H12A	0.9300
C8—H8B	0.9700	C5—H5A	0.9300
C7—H7A	0.9300	C15—H15A	0.9600
C14—C13	1.387 (2)	C15—H15B	0.9600
C14—H14A	0.9300	C15—H15C	0.9600
			
O1^i^—Co1—O1	180.0	O1—C6—C1	123.56 (13)
O1^i^—Co1—N1	86.99 (5)	O1—C6—C5	119.02 (14)
O1—Co1—N1	93.01 (5)	C1—C6—C5	117.41 (13)
O1^i^—Co1—N1^i^	93.01 (5)	C2—C3—C4	117.37 (14)
O1—Co1—N1^i^	86.99 (5)	C2—C3—C15	121.46 (17)
N1—Co1—N1^i^	180.00 (4)	C4—C3—C15	121.17 (16)
C7—N1—C8	115.04 (11)	C11—C10—C9	120.66 (14)
C7—N1—Co1	124.53 (9)	C11—C10—H10A	119.7
C8—N1—Co1	120.43 (8)	C9—C10—H10A	119.7
C6—O1—Co1	129.64 (11)	C5—C4—C3	122.29 (15)
C14—C9—C10	118.45 (12)	C5—C4—H4A	118.9
C14—C9—C8	123.16 (12)	C3—C4—H4A	118.9
C10—C9—C8	118.32 (12)	C12—C11—C10	120.60 (14)
C6—C1—C2	119.94 (13)	C12—C11—H11A	119.7
C6—C1—C7	120.59 (12)	C10—C11—H11A	119.7
C2—C1—C7	119.44 (13)	C12—C13—C14	120.58 (15)
N1—C8—C9	113.61 (10)	C12—C13—H13A	119.7
N1—C8—H8A	108.8	C14—C13—H13A	119.7
C9—C8—H8A	108.8	C13—C12—C11	119.04 (15)
N1—C8—H8B	108.8	C13—C12—H12A	120.5
C9—C8—H8B	108.8	C11—C12—H12A	120.5
H8A—C8—H8B	107.7	C4—C5—C6	120.84 (15)
N1—C7—C1	126.95 (13)	C4—C5—H5A	119.6
N1—C7—H7A	116.5	C6—C5—H5A	119.6
C1—C7—H7A	116.5	C3—C15—H15A	109.5
C9—C14—C13	120.66 (13)	C3—C15—H15B	109.5
C9—C14—H14A	119.7	H15A—C15—H15B	109.5
C13—C14—H14A	119.7	C3—C15—H15C	109.5
C3—C2—C1	122.15 (15)	H15A—C15—H15C	109.5
C3—C2—H2A	118.9	H15B—C15—H15C	109.5
C1—C2—H2A	118.9		

Symmetry codes: (i) −*x*+1, −*y*+2, −*z*.
